# Average Biomechanical Responses of the Human Brain Grouped by Age and Sex

**DOI:** 10.1007/s10439-025-03725-y

**Published:** 2025-04-09

**Authors:** Ahmed Alshareef, Aaron Carass, Yuan-Chiao Lu, Joy Mojumder, Alexa M. Diano, Olivia M. Bailey, Ruth J. Okamoto, Dzung L. Pham, Jerry L. Prince, Philip V. Bayly, Curtis L. Johnson

**Affiliations:** 1 Department of Biomedical Engineering, University of South Carolina, Columbia, SC USA; 2 Image Analysis and Communications Laboratory, Department of Electrical and Computer Engineering, Johns Hopkins University, Baltimore, MD USA; 3 The Military Traumatic Brain Injury Initiative, The Henry M. Jackson Foundation, Bethesda, MD USA; 4 Department of Radiology and Radiological Sciences, Uniformed Services University, Bethesda, MD USA; 5 Department of Radiology and Imaging Sciences, National Institutes of Health, Bethesda, MD USA; 6 Department of Biomedical Engineering, University of Delaware, Newark, DE USA; 7 Department of Mechanical Engineering, Washington University in St. Louis, St. Louis, MO USA

**Keywords:** Traumatic brain injury, Magnetic resonance imaging, Brain deformation, Group average, Finite element modeling

## Abstract

Traumatic brain injuries (TBIs) occur from rapid head motion that results in brain deformation. Computational models are typically used to estimate brain deformation to predict risk of injury and evaluate the effectiveness of safety countermeasures. The accuracy of these models relies on validation to experimental brain deformation data. In this study, we create the first group-average biomechanical responses of the brain, including structure, material properties, and deformation response, by age and sex from 157 subjects. Subjects were sorted intro three age groups—young, mid-age, and older—and by sex to create group-average neuroanatomy, material properties, and brain deformation response to non-injurious loading using structural and specialized magnetic resonance imaging data. Computational models were also built using the group-average geometry and material properties for each of the six groups. The material properties did not depend on sex, but showed a decrease in shear stiffness in the older adult group. The brain deformation response also showed differences in the distribution of strain and a decrease in the magnitude of maximum strain in the older adult group. The computational models were simulated using the same non-injurious loading conditions as the subject data. While the models’ strain response showed differences among the models, there were no clear relationships with age. Further studies, both modeling and experimental, with more data from subjects in each age group, are needed to clarify the mechanisms underlying the observed changes in strain response with age, and for computational models to better match the trends observed across the group-average responses.

**Supplementary Information:**

The online version contains supplementary material available at 10.1007/s10439-025-03725-y.

## Introduction

Traumatic brain injuries (TBIs) remain a serious societal concern, with over 1.7 million injuries occurring annually in the United States [1]. TBIs typically occur due to rapid head motion resulting from impacts in sports, automotive vehicle crashes, and falls [2]. The mechanical forces transmitted from the head to the brain have been a focus of research that aims to quantify the relationships between input loading, brain tissue mechanics, and acute pathophysiology [3–5]. These relationships are critical for developing better injury prevention technologies and injury prediction models needed in contact sports, military operations, motor vehicle use, and other daily activities [6–8].

Anatomically detailed computational models or physical surrogates of the brain that predict tissue mechanical response are crucial for mechanistically understanding brain injury and developing injury mitigation countermeasures [9–11]. While there have been recent innovations in subject-specific brain modeling [12–14], most of the computational models used for TBI prediction and prevention are generated with a generic geometry to represent the response of a specific gross anthropometry (e.g., a 50th percentile male) [9, 11]. Further details on the development and evaluation of computational brain models can be found in the referenced literature [9, 10]. Models that represent the average properties of a population are needed because subject-specific properties are not always available, and it can be computationally intensive to run a large number of individual simulations for a large cohort. These average models are important in the injury prevention and safety design fields, since they can capture broader ranges of individuals with relative computational efficiency and can focus on key parameters that determine overall response. Applications like vehicle safety testing and helmet design have used computational models with age, sex, and anthropometric cohort stratifications, such as the Total Human Model for Safety (THUMS) model [15]. However, the head and brain regions of these models are scaled from the 50th percentile male model based on whole body mass or stature [16]. This scaling is similar to the creation of population and sex specific anthropometric test devices (ATD) [17] used to evaluate helmet efficacy and automotive safety. To date, there is a lack of brain templates for biomechanical modeling that accurately characterize populations based on demographic variables with respect to geometry and material properties.

An additional challenge in brain model development is the evaluation of model accuracy in predicting the brain’s biomechanical response against experimental human brain deformation data [18]. This is especially difficult when creating subject-specific or population average response models for specific demographic groups, such as sex and age stratified cohorts, primarily due to limited experimental data. Traditional datasets of brain deformation for model evaluation are from post-mortem human subjects at concussive loading conditions. They have a relatively small number of specimens, typically older in age, due to the complexity of the experiments and limited availability of cadaveric tissue [19, 20]. The brain deformation response of models is primarily evaluated against such high-rate cadaveric data [19–22] which quantify brain deformation using bi-planar X-ray or sonomicrometry, in addition to low-rate in vivo [6, 23–25] tests using magnetic resonance imaging (MRI). Comparisons are often made to subjects with the closest anthropometry or target population characteristics, which does not consider the natural variation among individuals, or using simple geometric or mass scaling to match the model to the subject [26].

Alternatively, in vivo experiments on healthy human volunteers at non-injurious loads using advanced magnetic resonance imaging (MRI) techniques offer a rich source of data on a wider range of individuals. Magnetic resonance elastography (MRE) utilizes small, harmonic head motion to estimate in vivo material properties [27, 28], and tagged MRI (tMRI) uses repeated, impulsive head motion to measure brain deformation [23, 25]. In combination with structural MRI scans, the MRE and tMRI imaging techniques generate the geometry, material properties, and brain deformation response needed to build, parameterize, and evaluate a computational brain model for individual subjects or for a target cohort of individuals [12, 29].

As part of an ongoing project that provides a publicly shared dataset, our group has conducted scans on more than 150 subjects spanning a large age range across both sexes [6]. The dataset includes structural MRI and either MRE or tMRI in a subset of subjects. This data has been used extensively to build and evaluate computational models [12, 29, 30], quantify brain natural frequencies [31, 32], understand brain mechanical vulnerability to direction of head motion [33], correlate brain deformation to acute and chronic TBI spatial patterns [34], and understand subject-specific differences in brain deformation response [35–38]. The goal of this study is to create the first group-average responses by age and sex from this large brain biomechanics dataset. The first objective is to compute and compare group-average anatomical templates, material properties, and maximum strain templates for the young, mid-age, and older adult brains for the male and female populations. The second objective is to build and simulate group-average finite element (FE) models using linear viscoelastic material properties calibrated from MRE to investigate differences in biomechanical responses across the groups.

## Materials and Methods

### Subject Information and MRI Acquisition

Imaging data were collected in healthy adults at three sites as part of a multi-institutional effort aimed at generating measurements of the in vivo biomechanical response of the human brain [6]. All images were collected after subjects provided informed consent under separate institutional review board (IRB) protocols approved by the Washington University in St. Louis, the University of Delaware, and the Clinical Center at the National Institutes of Health, and processed under an IRB protocol at the Johns Hopkins University. Anatomical scans were collected at all three sites, while high-resolution MRE scans were acquired at the University of Delaware. Tagged MRI scans were conducted in the Clinical Center at the National Institutes of Health. In total, scans from 157 volunteers (78 male/79 female; 18–73 years old) were collected using anatomical MRI sequences and either MRE or tMRI at the time of writing and included in this analysis (Table 1). Site-specific imaging protocols, sequence parameters, and data processing are described in an overview article [6] and in a publicly available data repository [39].

**Table 1:**
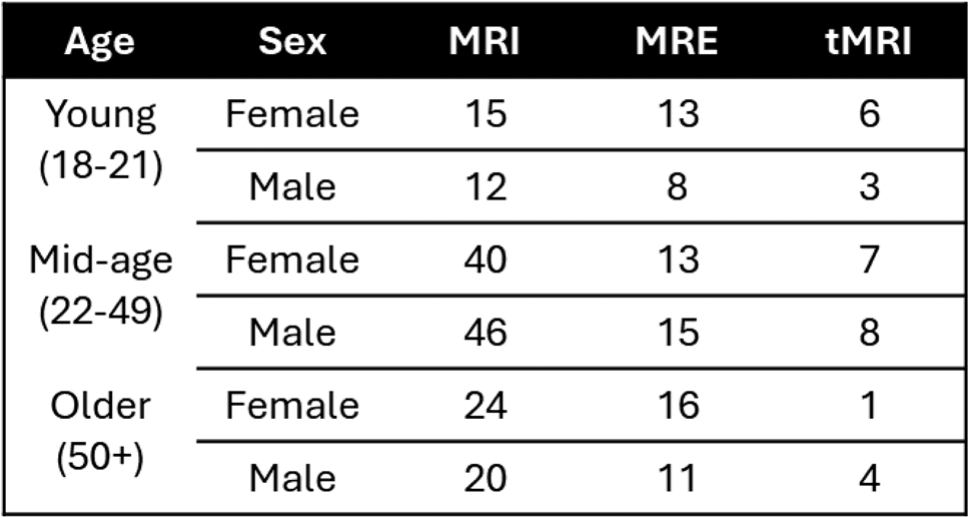
Number of subjects with anatomical MRI, MRE, and tMRI imaging data

### Data Processing

Structural MRI scans were collected for every subject at every site, and included T1-weighted (T1w), T2-weighted (T2w), and diffusion weighted images (DWI). Additional scans including susceptibility weighted imaging (SWI), time of flight (ToF), and FLAIR were acquired on a subset of the cohort, but these images were not used in this study. Anatomical images were processed to provide voxelwise segmentation maps of various anatomical structures and estimate white matter diffusion properties. For anatomical segmentation, the T1w and T2w images were rigidly registered to the MNI-152 space [40] using the Advanced Normalization Toolkit (ANTs) v2.2 [41], resampled to 0.8 mm isotropic resolution, and processed with a deep learning-based segmentation algorithm (Spatially Localized Atlas Network Tile—SLANT [42]) to generate 132 brain labels, including a brain mask and dura mask. The falx and tentorium were also automatically segmented for each subject using a fast-marching, multi-atlas-based segmentation based on the T1w and either the T2w or SWI images [43].

High-resolution MRE data were acquired using a 3D multiband, multishot spiral MRE sequence with OSCILLATE acceleration [28, 44]. The MRE acquisition included three separate scans at 30, 50, and 70 Hz, with 1.5 mm isotropic imaging resolution. MRE scans encode displacements generated through occipital head actuation using the Resoundant pneumatic actuator system with a soft “pillow” driver (Resoundant Inc., Rochester, MN, USA). All MRE imaging data were acquired on a Siemens 3T Prisma scanner with a 64-channel head/neck coil. The brain tissue material properties from MRE were estimated using the nonlinear inversion algorithm (NLI) [45]. NLI utilizes an inverse finite element-based iterative optimization routine to estimate material properties of brain tissue. NLI models the tissue as a heterogeneous, linear viscoelastic solid and returns whole-brain maps of the complex shear modulus, G* = *G’* + i*G’’*, where *G’* and *G’’* are the shear storage and loss moduli, respectively. The shear stiffness (μ = 2|G*|^2^/(*G’* + G*) represents the viscoelastic resistance of the brain to mechanical load, while the damping ratio (ξ = *G’’*/2*G’*) represents the relative viscous-to-elastic behavior of the brain tissue. The material property maps were rigidly registered to the processed T2w images of each subject and linearly interpolated to 0.8 mm isotropic resolution to match the segmented T1w images and SLANT output.

Tagged MRI was used to acquire 3D dynamic brain deformation. A custom MRI-compatible device was used to facilitate a mild, repeatable head axial rotation around the neck (~3 rad/s, 200 rad/s^2^). Head kinematic angular velocity and acceleration were measured using an MRI safe rotational encoder (Microner Inc., Camarillo, CA, USA). Brain motion was imaged using a 1:1 spatial modulation of magnetization (SPAMM) sequence with a multi-slice acquisition that incorporates tags along three orthogonal directions to provide full brain coverage at 18-20 ms temporal resolution and 8 mm tag spacing, using fewer than 150 repetitions per subject. All tagged MRI data were acquired on either a Siemens 3T Biograph mMR or Siemens 1.5T Aera scanner with a combination of flexible array and spine receive coils to accommodate the head kinematic device within the bore of the scanner. The tagged images were initially resampled to a 1.5 mm isotropic resolution and the 3D displacements for each voxel were computed using the Harmonic Phase—Finite Element method (HARP-FE) [46]. Lagrangian strain was then computed directly as the spatial derivative of the displacement field. Additional information on the tagged MRI acquisition and processing can be found in the following references [23, 47]. The maximum principal strain (MPS) of each voxel was computed as the first eigenvalue of each strain tensor. The max MPS over the entire strain time-history was chosen as the metric of interest when creating group-average templates. The scalar MPS maps were rigidly registered to the processed T1w images of each subject and linearly interpolated to 0.8 mm isotropic resolution to match the segmented T1w images and SLANT output.

### Anatomical Template

**Fig. 1:**
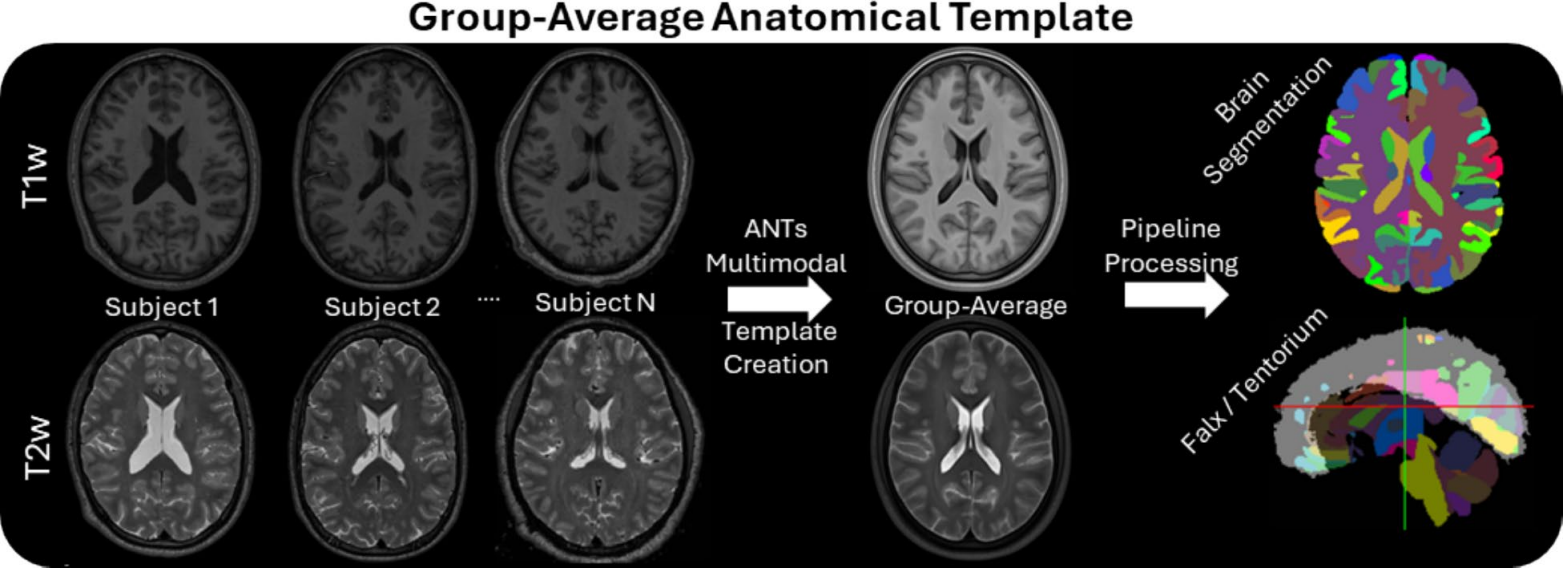
Overview of the anatomical template creation process. The average T1w and T2w template images are created for each group using the ANTs multimodal template creation script. The average images are then segmented using automated algorithms with a direction orientated thinning to enhance the realism of the falx and tentorium.

The ANTs template creation script with multimodal input was used to create average T1w and T2w images and brain segmentations (Figure 1). The rigidly aligned images in MNI space from Sect. “Data Processing” at 0.8 mm isotropic resolution were used. Briefly, the template script calculates an average anatomical template through iterative nonlinear registrations and averaging, followed by a Laplacian sharpening filter to enhance edges. Additional information on the open source script can be found in the referenced literature [48, 49].

The average T1w and T2w template images were then processed in the same manner as the individual subject images in Sect. “Data Processing.” The brain mask, dura mask, and 132 brain labels were generated using SLANT. The brain volumes of the template images were computed using the SLANT image and compared to the volumes of the input subject brains. The automated falx and tentorium algorithm, however, did not provide a qualitatively accurate segmentation, likely due to blurriness at the cortical regions in the template images. To create the falx and tentorium for the template anatomy of each group, the individual falx and tentorium masks of each subject were nonlinearly warped to the template T1w with nearest neighbor interpolation. For the falx, we create a union of all the warped falxes and then the group-average falx was thinned to be no more than two voxels thick in the left-right plane. We follow a similar procedure for the tentorium, with the thinning constraint being in the inferior-superior plane. Statistical comparisons of brain and ventricle volumes among the groups were performed using a two-way ANOVA with age and sex as categorical variables. If age is found to be significantly different among the groups (*p* < 0.05), a post-hoc multiple comparisons test is conducted to determine which groups are significantly different.

### MRE Material Property Template

**Fig. 2:**
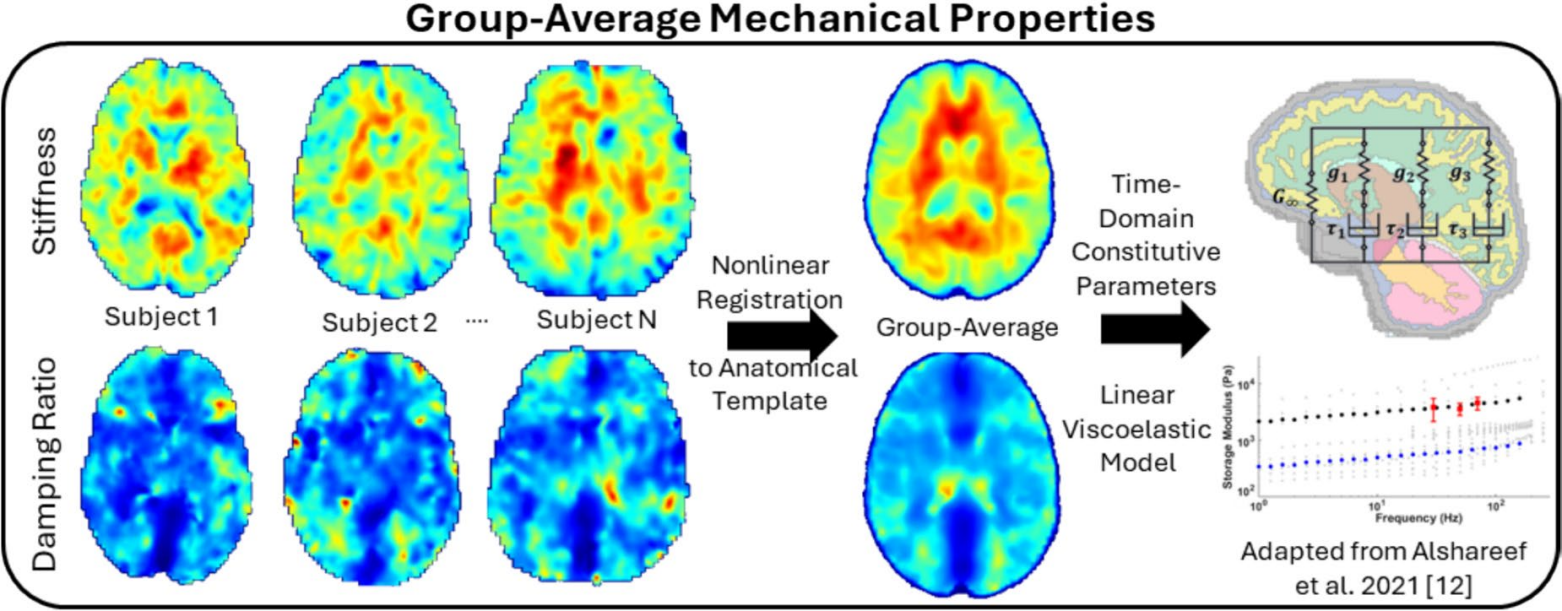
Generation of the average MRE material properties for each group. ANTs nonlinear registration was used to warp all individual properties to the template anatomical space. Group-average material properties were calculated using a voxelwise average with a majority vote mask to remove edge voxels. Time-domain material properties were computed using a previous parameterization [12].

MRE data were used to generate average material properties for each of the six template anatomies, by age and sex. To generate the average material property map for each group, the MRE magnitude image of each subject (1.5 mm isotropic resolution) was nonlinearly registered using ANTs to the group T2w anatomical template (Fig. 2). The spatial transformations were then applied to warp the scalar storage modulus, loss modulus, shear stiffness, and damping ratio maps of each subject at all three MRE head actuation frequencies (30, 50, 70 Hz) with linear interpolation. The average property at each voxel was defined as the average of all subjects within the group, with a standard deviation map also calculated. A majority (50%) vote was used to define the MRE mask and remove any edge voxels that contain property values for fewer than half the number of subjects.

The average MRE properties for each group were then used to compute regional, time-domain linear viscoelastic (LVE) material properties for each group. The time-domain properties were computed using a combination of the average in vivo data at 30, 50, and 70 Hz, and ex vivo data from mechanical tests on excised brain tissue from animal and human specimens [50]. The parameterization technique involved scaling the ex vivo to match the in vivo MRE data at the limited 30–70 Hz frequency range, and then fitting the linear viscoelastic constitutive model parameters to the scaled ex vivo and MRE in vivo data using the full frequency range for each brain region. The SLANT regions were merged to form six regions: the cerebrum white matter, cortical gray matter, deep gray matter, cerebellum white matter, cerebellum gray matter, and brainstem. The model parameters include the instantaneous shear modulus (${G}_{0}$), infinite shear modulus (${G}_{\infty }$), the Prony series fractional contributions (${g}_{i}$), and Prony series time constants (${\tau }_{i}$). Each fit is constrained for up to 3 Prony series terms. A detailed explanation of the parameterization can be found in Alshareef et al. [12].

Statistical comparisons of median brain stiffness and damping ratio for all MRE frequencies were performed in the same manner as described in Sect. “Anatomical Template.”

### Tagged MRI Strain Template

The tMRI experiments result in 4D data (3D space and time) for each of the three displacement component (${d}_{x}, {d}_{y}, {d}_{z}$) and each of the six strain components (${E}_{xx}, {E}_{yy}, {E}_{zz}, {E}_{xy}, {E}_{xz}, {E}_{yz}$). For this high-dimensional tensorial data, the nonlinear warping process of the registrations used to create the average templates can lead to errors in tensor orientation and temporal evolution. To simplify this analysis to a scalar value, the peak maximum resultant displacement (MRD) and maximum principal strain (MPS) across the time-history for each voxel were chosen as a representative measure of brain deformation. MPS has been used extensively in the brain biomechanical modeling literature [9, 11] and has been shown to be predictive of TBI risk [4, 5, 51–53]. To transform the MPS, a similar registration scheme as used for the MRE data was implemented (see Sect. “MRE Material Property Template”). For each subject, the peak MPS was calculated, and a 3D strain image was generated in the subject space. The average tMRI strain templates were created for the three age groups, combining both male and female subjects, due to a small sample size in the young and older adult groups. This choice was informed by previous work showing no statistically significant differences between male and female tMRI responses for similar loading conditions [47]. To generate the average strain map for each group, the T1w image in the tMRI space (1.5 mm isotropic resolution) was nonlinearly registered using ANTs to the group T1w anatomical template. The spatial transformations were then applied to warp the peak MRD and MPS maps of each subject with linear interpolation. The average strain at each voxel in the template space was defined as the average of all subjects within the group, along with a standard deviation calculation. A majority vote was used to define the strain mask in the same manner as Sect. “MRE Material Property Template.”

Statistical comparisons of the MRD and MPS metrics were performed using a one-way ANOVA with age group as a categorical variable. A post-hoc comparison was conducted if a significant difference was found ($p<0.05$).

### Group-Average Computational Models

Using the template geometry and average material properties for each of the six groups, a computational FE model was created in LS-Dyna (Ansys Inc., Canonsburg, PA, USA) directly from the segmented MRI images using a custom pipeline. This pipeline was previously used to generate, parameterize, and evaluate the strain response of subject-specific brain models from MRI, MRE, and tMRI data [12]. A refined segmentation was first created in the pipeline using the combined labels of the six brain regions and the ventricles, including the lateral, third, and fourth ventricle labels. The subarachnoid space (SAS) was preliminarily defined as any voxel within the dura mask but not within the brain or the falx and tentorium masks. The skull was defined as a layer of voxels outside the SAS to encompass the cranium. A refinement step was conducted for the SAS to ensure that all brain voxels had at least one layer of SAS voxels between the brain and skull to computationally simulate the fluid layer. The falx mask was then refined to connect in the superior direction to the skull. To ensure a non-enclosed spinal cord, any voxels in the superior-inferior direction below the brainstem are relabeled so that SAS voxels become brainstem and skull voxels become foramen. In total, the model contained 11 parts, with the six brain regions, ventricles, SAS, falx/tentorium, foramen, and skull. Each model was created by generating solid, constant-stress hexahedral elements from each voxel in the refined segmentation. The mesh contains elements with 1.5 mm isotropic length across the brain with non-smoothed edges. All voxels have shared nodes between all regions of the brain and skull. This direct voxel-based FE method has been used to successfully generate brain models previously [13, 54] with little difference in computed global maximum principal strains as compared to models with smoother boundaries [14].

The FE model brain material properties were assigned using the group-average linear viscoelastic material properties derived from MRE data as described in Sect. “MRE Material Property Template.” All other material properties, including the falx/tentorium, foramen, ventricles, and skull, were obtained from previous mechanical tests on these tissues or previous computational brain models [53, 55–57] and are provided in a previous study [12]. The SAS was treated as soft viscoelastic material using solid hexahedral elements with shared nodes in neighboring regions. The material properties of this space were modeled using a linear viscoelastic model with $\rho =1.04 g/c{m}^{3}$, $K=2.19 GPa$, ${G}_{0}=0.5 kPa, {g}_{1}=0.8, {\tau }_{1}=12.5 ms$, as defined in previous models [12]. The choice of model and properties was chosen based on previous modeling efforts that evaluated model results against low-severity in vivo brain deformation and high-severity cadaveric brain displacement experiments [12, 30, 53]. All group-average models were simulated under a single neck rotation from one representative tMRI subject (S01-NR from Alshareef et al. [12]). This kinematic profile was chosen to investigate the difference in brain deformation among the group due to the same severity and duration of loading, though each subject’s head kinematics may play a role in observed variances in the tMRI group-average results. The kinematic loading time-history was to the rigid skull. The models were simulated for a duration of 160 ms on an eight-core machine using the LS-Dyna serial 10.0 solver with double precision.

## Results

### Group-Average Anatomical Template

**Fig. 3:**
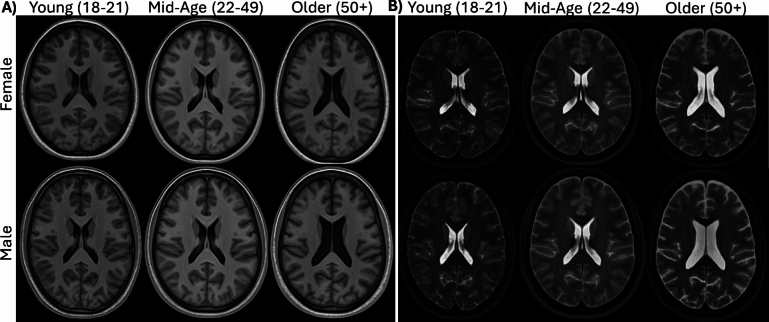
Anatomical templates for the six groups for a total of 157 subject scans, for **A** T1-weighted and **B** T2-weighted images. Table 1 contains information about the number of subjects within each group

**Fig. 4:**
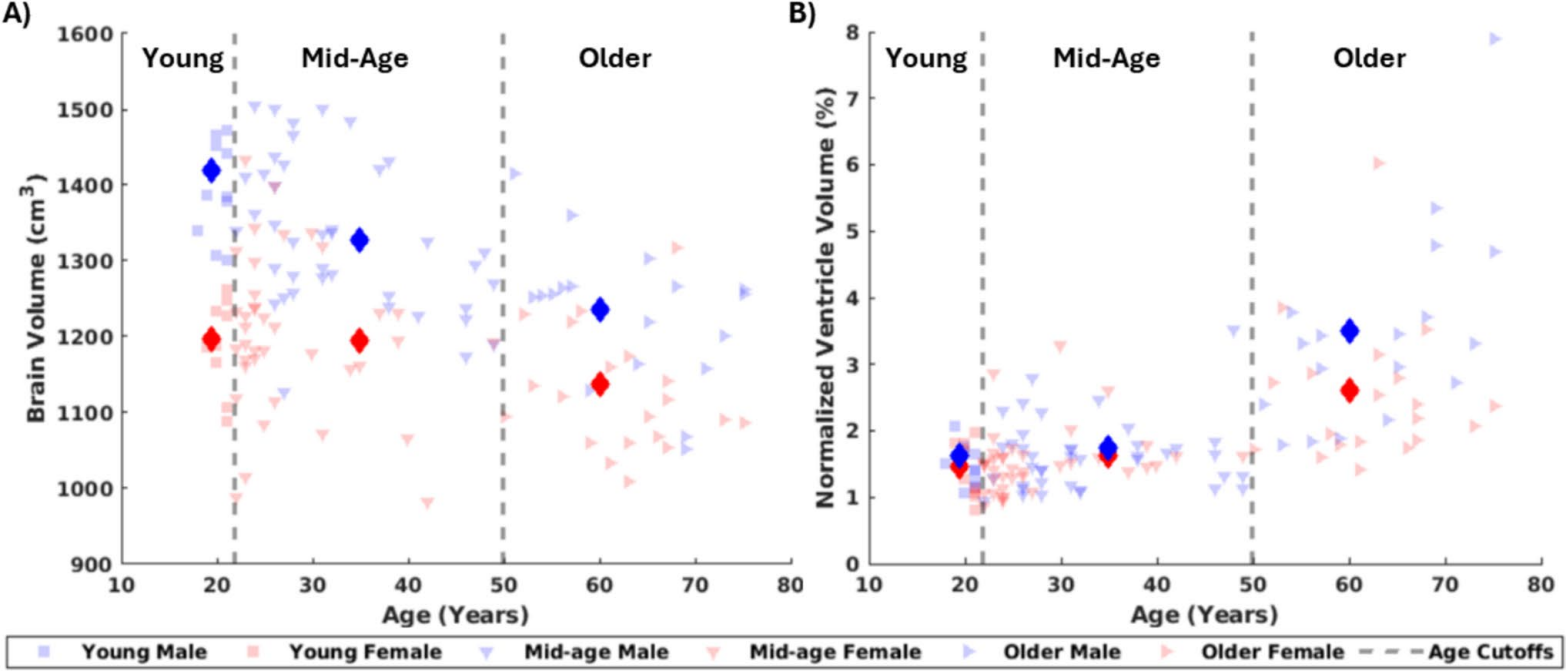
**A** Brain segmentations were used to compute the brain volume for each group-average template for female (red) and male subjects (blue). The black dashed lines represent the age cutoffs for each group. The semi-transparent markers depict the subjects used to make each group-average template, while the solid diamond markers depict the group-average neuroanatomy. **B** The normalized ventricle volume percentage was also computed to compare among groups and the input subject data, and is presented in the same fashion.

T1w and T2w group-average template images were created for each of the six groups and are depicted in Fig. 3. There are qualitative differences in the average head and brain geometry of each group, primarily the volume of the lateral ventricles, thickness of the subarachnoid space, and depth of the sulcal spaces. These differences across the age group stratifications are an expected pattern of cerebral atrophy [58]. A quantitative comparison of the brain and ventricle volumes of the average templates versus the subjects within each group are presented in Fig. 4 and Table 2. The average of the volumes calculated from the segmentations of the individual subjects match well with the group-average template volumes, with higher standard deviations computed for the older groups. Males have a larger brain volume across all age groups, which is expected [59]. Once normalized for brain volume, there is little sex difference in the ventricle volumes, except in the older adult group where males have larger ventricles even after normalization, indicative of greater atrophy. The older adult group also exhibits larger standard deviations in ventricle volume, likely due to the variability of cerebral atrophy rates during healthy aging in the age range (51–77 years) of this group [60]. Statistical tests supported these trends and showed significant differences with age and sex (*p* < 0.05) for brain volume, ventricle volume, and normalized ventricle volume. The post-hoc comparisons indicate that the older group differs significantly from the other age groups, while the young and mid-age groups do not differ significantly.

**Table 2:**
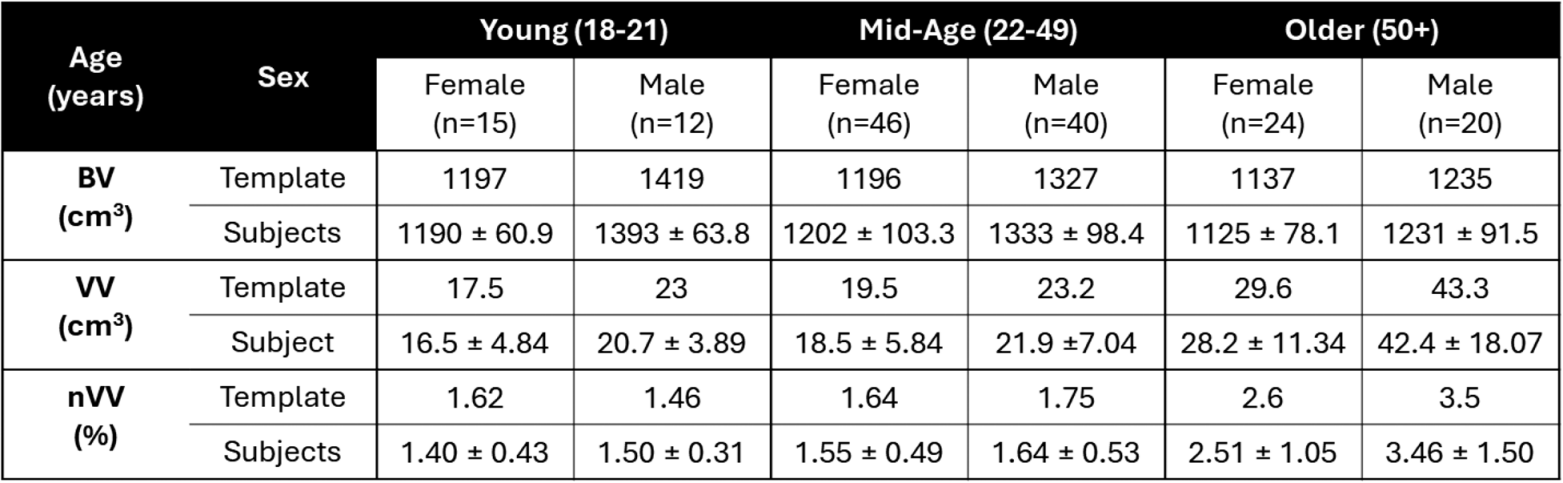
Comparison of the brain volume (BV), ventricle volume (VV), and normalized ventricle volume (nVV) for the six group templates versus the average and standard deviation of the subjects within each group.

### Group-Average Material Properties

**Fig. 5:**
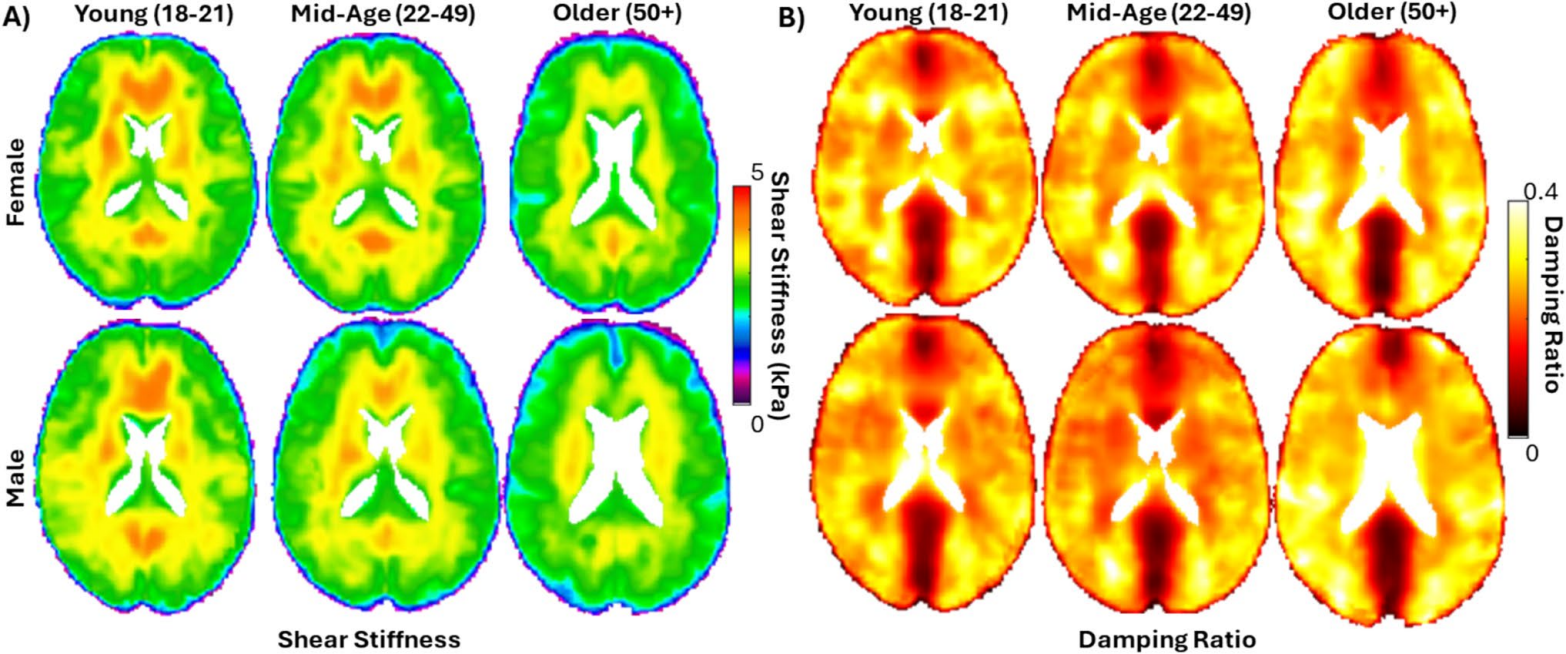
Average material properties were computed for the six groups, including **A** shear stiffness and **B** damping ratio, and are depicted for an axial slice of the brain from MRE experiments at 50 Hz head actuation

Average in vivo brain material properties for each group were computed from MRE experiments at 30, 50, and 70 Hz harmonic actuation of the head. The voxelwise average shear stiffness and damping ratio from 50 Hz MRE experiments are presented in Fig. 5. Qualitatively, the average results of the young and mid-age groups are similar in spatial distribution and magnitude, and match previous atlas of average material properties from a large collection of MRE datasets [61]. The older adult group shows distinctly lower average shear stiffness values, which is an expected trend in the aging brain [62–64].

**Fig. 6:**
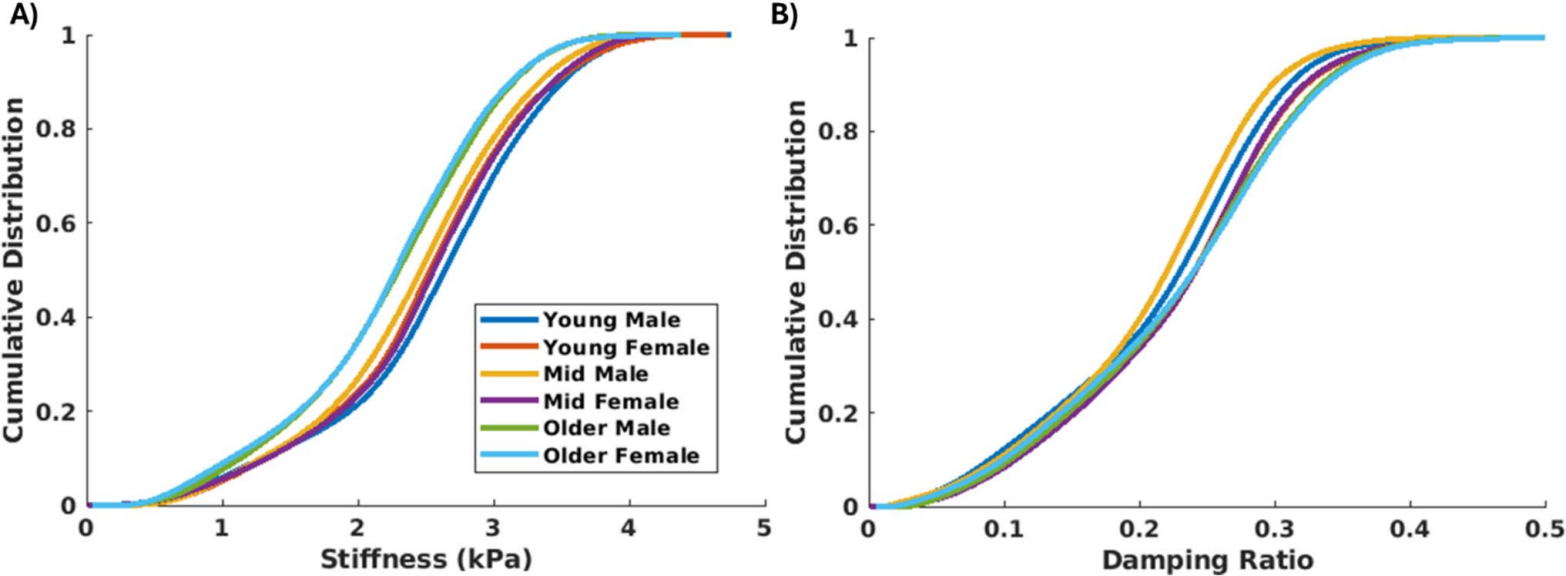
Distribution of **A** shear stiffness and **B** damping ratio for the six group-average material property templates from MRE experiments at 50 Hz head actuation

A cumulative distribution of 50 Hz shear stiffness and damping ratio are presented in Figure 6. The cumulative distribution of the 50 Hz shear stiffness for the older adult group are clustered separately and at lower stiffnesses than the young and mid-age groups. The damping ratio showed slight differences, but there was no trend with sex or age. The 30 Hz and 70 Hz data had similar trends for shear stiffness and damping ratio. A quantitative comparison of the median material properties for the group-average versus individual subjects for all three frequencies of MRE actuation is provided in Table 3. The median properties of the template were similar to the subjects for all groups, falling within one standard deviation of the subject average. Statistical tests using the median properties for all subjects show a significant difference with age ($p<0.05$) for brain stiffness across all frequencies (30, 50, and 70 Hz), but only at 50 Hz and 70 Hz for the damping ratio. Differences by sex are also statistically significant ($p<0.05$) at 50 Hz and 70 Hz, but not at 30 Hz. Post-hoc comparisons indicate that only the older adult group differs from the other groups in all comparisons, exhibiting lower stiffness and higher damping ratio.

**Table 3:**
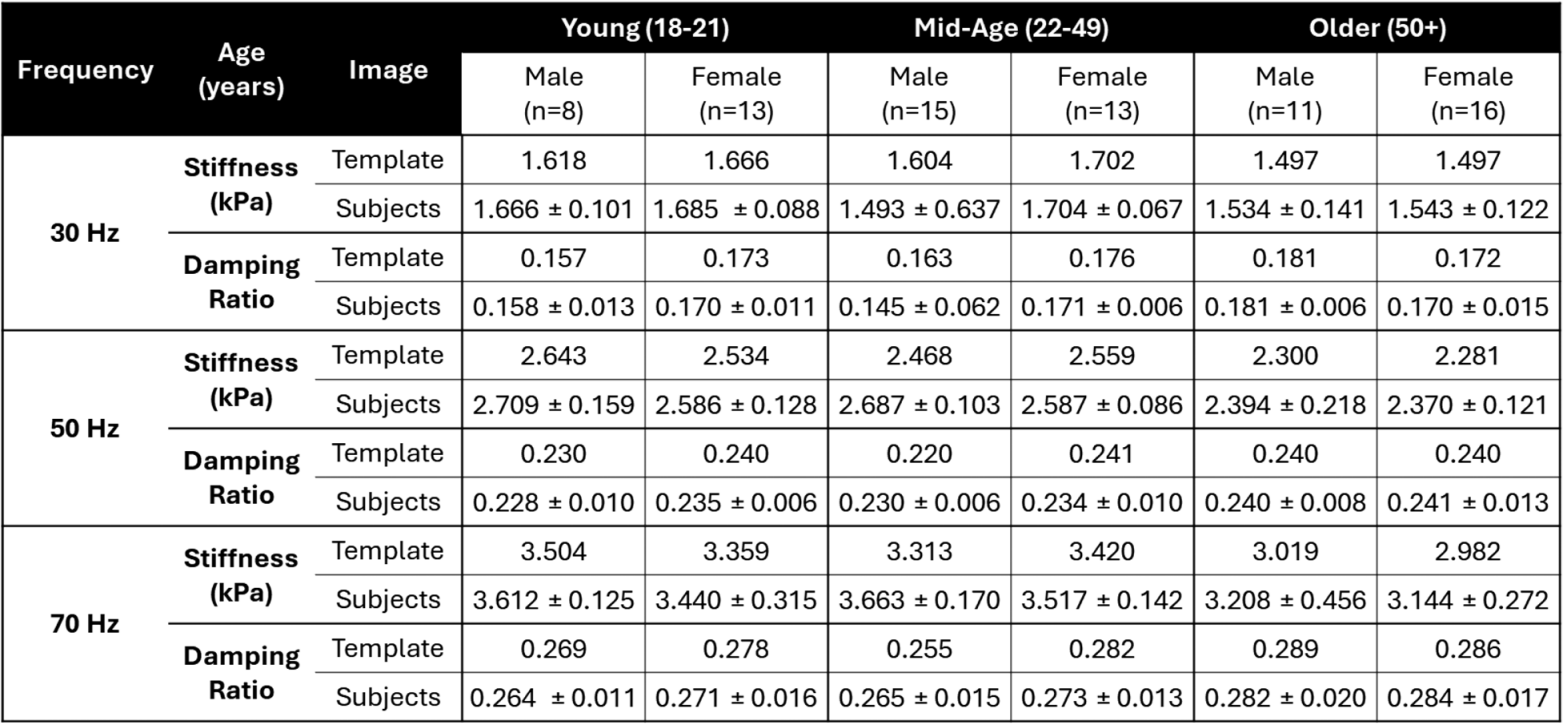
Comparison of the median MRE stiffness and damping ratio values for the whole brain for each of the six group templates versus the average and standard deviation of the whole-brain median for the subjects within each group

### Group-Average tMRI Strain Response

**Fig. 7:**
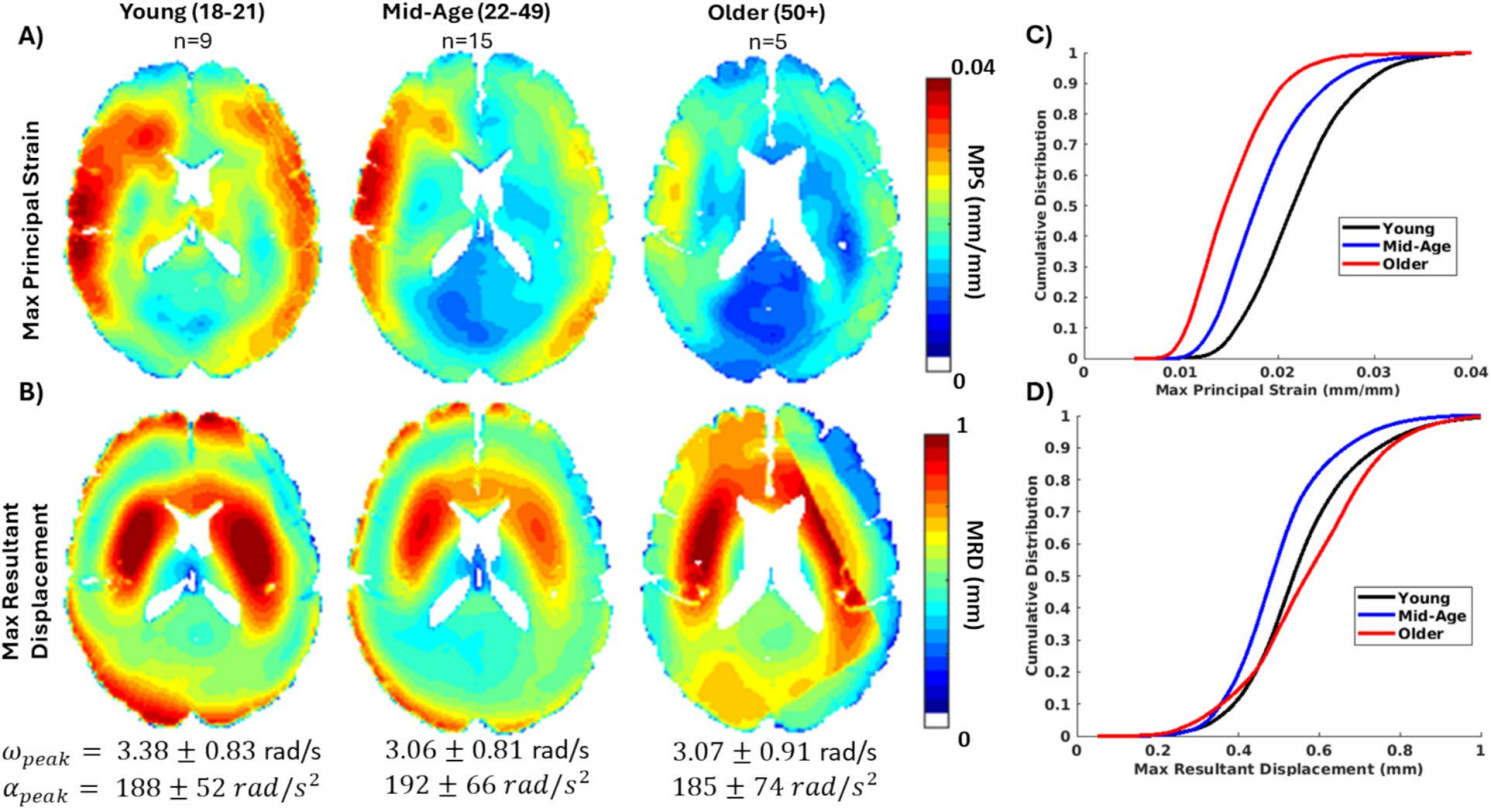
The average **A** maximum principal strain (MPS) and **B** maximum resultant displacement (MRD) from tagged MRI experiments were computed for the three age groups and are shown for an axial slice of the brain. The average and standard deviation of the peak angular acceleration (${\alpha }_{peak}$) and angular velocity (${\omega }_{peak})$ for all subjects within each group are presented below the brain slices. Note that discontinuities in the top right portion of the MRD and strain fields for the older group-average resulted from an inconsistent MRI field of view (i.e., missing data) during acquisition. **C** Distribution of maximum resultant displacement (MRD) and **D** maximum principal strain (MPS) for the three age groups from the tagged MRI head rotation experiments.

The average brain deformation response to non-injurious impulsive loading was computed for each group from tMRI neck rotation experiments. The voxelwise average MRD and MPS for the three age groups, combining both male and female subjects within each group, are presented in Fig. 7, in addition to a cumulative distribution of the MRD and MPS. Note that discontinuities in the top right portion of the MRD and strain fields for the older group-average resulted from an inconsistent MRI field of view (i.e., missing data) during acquisition. Removing these artifacts did not significantly change the distribution of the MRD and MPS data. Though it is difficult to compare the strain fields from individual subjects due to differences in the applied head rotation magnitude, the average responses had similar peak head angular velocity and acceleration. Qualitatively, the average results of the young and mid-age groups are similar in spatial distribution of MPS and MRD, with notable left-right asymmetry in the MPS response. There is slightly lower displacement and strain in the cortical regions of the mid-age group relative to the young group. The cumulative distribution of MPS and MRD show consistently lower displacement and strain with the increasing age of the groups. This trend is also reflected in the 50th and 95th percentile MPS and volume fraction of MPS metrics (Table 4), but not in the MRD metrics. The older group had lower 95th percentile MPS but a similar 95th percentile MRD to the other groups. Statistical comparisons showed no significant differences among the age groups were found for the 50th and 95th percentile MRD. Significant differences ($p<0.05$) were observed for the 50th percentile MPS and VF MPS > 0.01 and 0.02. Post-hoc comparisons revealed significant differences between the older and younger group, but not the mid-age group. A marginal level of statistical significance was observed for the 95^th^ percentile MPS ($p=0.0964$) and VF MPS > 0.03 ($p=0.0751$).

**Table 4:**
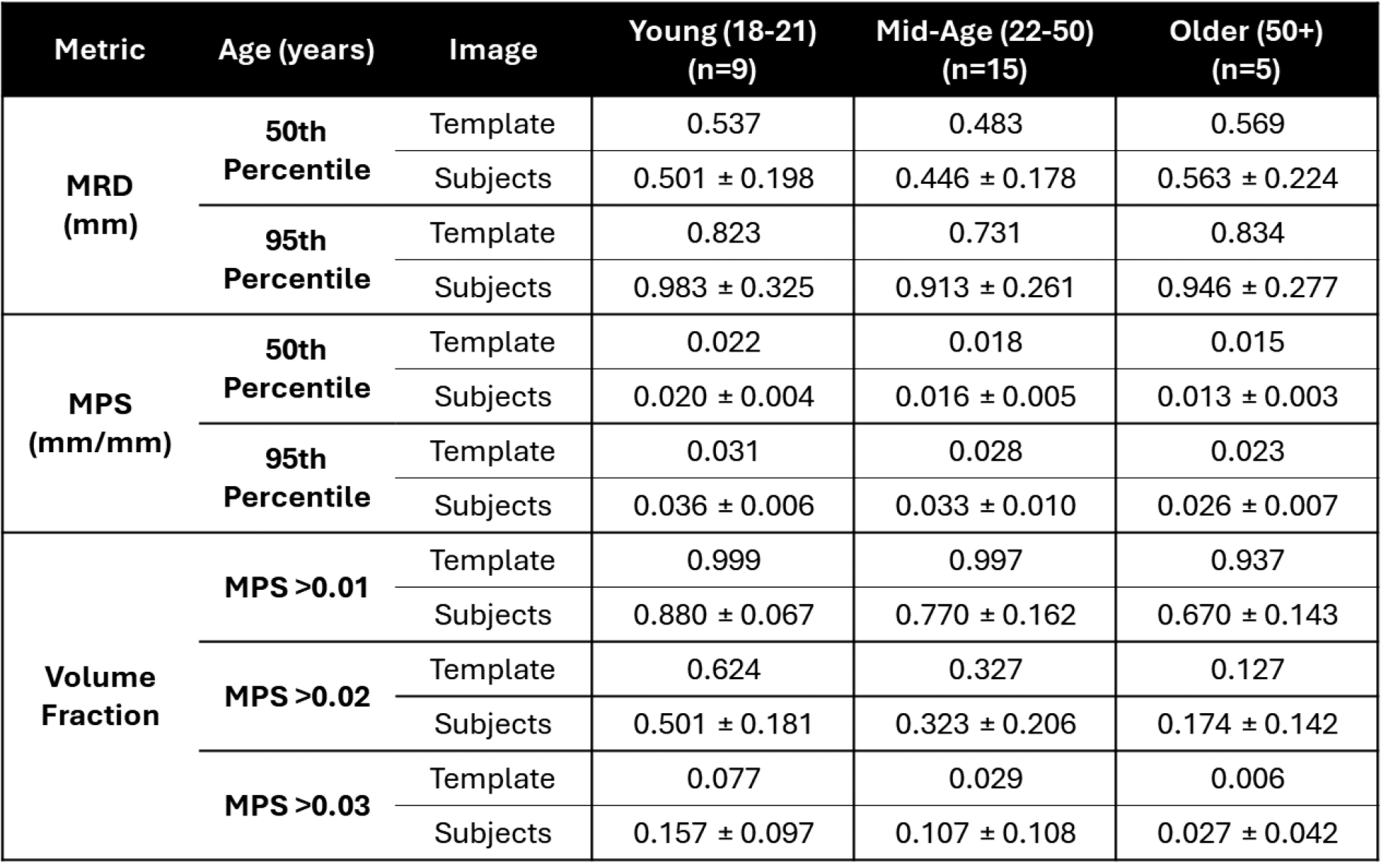
Comparison of the 50th percentile (median) and 95th percentile brain deformation metrics from the tagged MRI head rotation experiments, including maximum resultant displacement (MRD), maximum principal strain (MPS), and volume fraction of MPS

It is also notable that unlike the volume or material property metrics, the group-average 95^th^ percentile MRD and MPS metrics, and VF > 0.03 were consistently lower than the average of the subjects. This is likely due to the higher variability of the experimental loading conditions, including the magnitude of applied acceleration and variability in the positioning of the head, of the tMRI tests and higher subject-to-subject variability in the locations of maximum strain. The voxelwise standard deviation of MRD and MPS are shown in supplementary Figure S1, showing higher variation in the displacement field but not the strain field for the three age groups.

### Group-Average Computational Model Strain Response

**Fig. 8:**
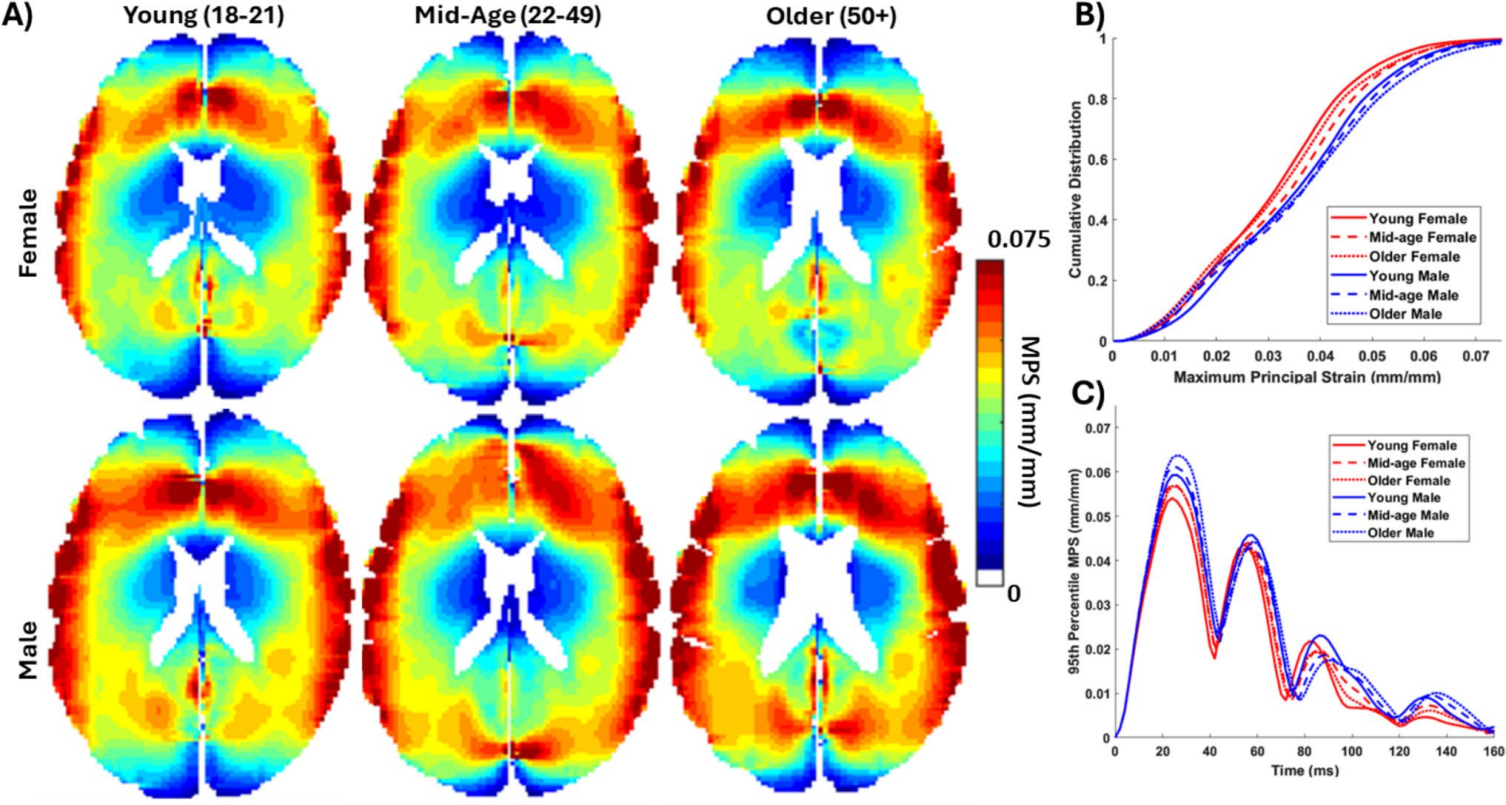
FE model simulations using common head kinematics loading conditions from tagged MRI experiments are shown for an axial slice of the brain for all six group-average models. **A** The maximum principal strain (MPS), **B** distribution of MPS, and **C** time-history of the 95th percentile MPS from the simulations of the six group-average FE models

Six group-average computational models were generated using the template geometries, parameterized with a linear viscoelastic constitutive model using MRE data for each group, and simulated with the same representative tMRI neck rotation loading condition. The spatial distribution of MPS for an axial slice of the model are shown in Fig. 8, in addition to a cumulative distribution plot of the MPS and a 95th percentile MPS time-history. All the models showed similar strain spatial distribution, cumulative distribution, and 95th percentile time history, with slight differences in the peak MPS with age and sex. The spatial distribution of strain was also left-right symmetric, unlike the tMRI group-average results.

In a quantitative comparison of the MPS metrics (Table 5), the models generally resulted in higher strains than the group-averaged tMRI results. There is little dependence on sex or age among the models, with much smaller differences among the brain deformation response of the group models than those of the MRE or tMRI results.

**Table 5:**
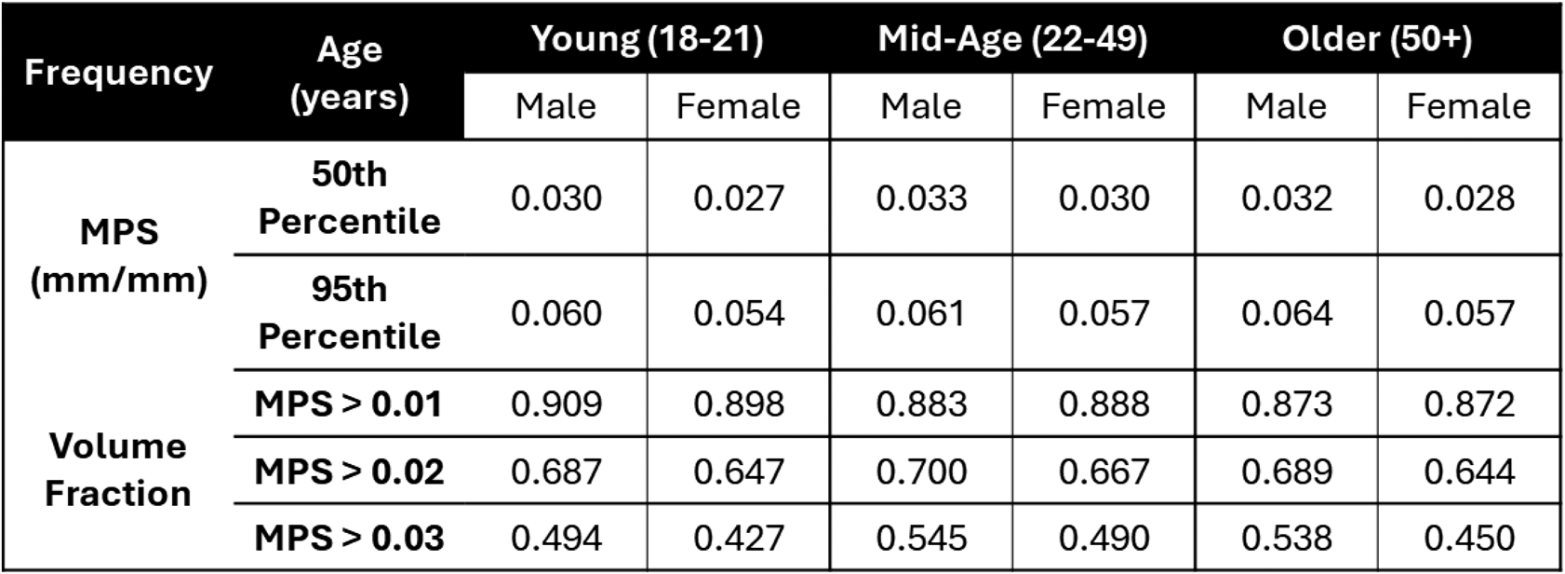
Comparison of the 50th percentile (median) and 95th percentile brain deformation metrics from the tagged MRI head rotation experiments, including maximum resultant displacement (MRD), maximum principal strain (MPS), and volume fraction of MPS

## Discussion

The characterization of the brain’s biomechanical response to head motion is crucial for understanding the relationship between head impact and the pathophysiology of TBI. This relationship underpins the accuracy of predicting injury from head impact and developing improved safety countermeasures. A primary tool in this effort is modeling with both computational and physical models, which estimate brain deformation for a given impact. Evaluation of the accuracy of these models depends on the availability of experimental data on human brain deformation. Datasets of brain biomechanics are scarce and usually include a limited number of subjects, hindering group-average analysis and FE model evaluation. Though there have been advances in the creation and evaluation of subject-specific computational models, models built to represent certain groups or populations have not been evaluated against experimental brain deformation data. This is the first study to generate group-average structural and biomechanical response of the brain, separated by age and sex. The group-average responses include representative neuroanatomy from 157 subjects using structural MRI, average material properties from 77 subjects using MRE, and average brain deformation responses from 29 subjects using tMRI. Group-average FE models were created and simulated to compare the experimental group-average brain deformation trends to those predicted by the models.

### Group-Average Neuroanatomy and Material Properties

The group-average anatomical templates showed a dependence on both age and sex. Qualitatively, older adult brains had progressively larger ventricles, wider sulci, and more fluid in the subarachnoid space. The brain volume for the males was consistently higher than females but showed a decrease across all three age groups. The female brain volume was consistent between the young and mid-age groups, with a similar rate of atrophy in the older adults. The older adults, for both male and female, also exhibited higher variability as seen in the normalized ventricle volume plot (Fig. 4B) and quantitative metrics (Table 2). This variability in atrophy may be an important factor in the biomechanical response of the brain, as atrophy can alter the amount of brain deformation [65].

The group-average shear stiffness from MRE showed a distinct trend in the older adult group with both females and males exhibiting approximately 7-10% lower stiffness as compared to the younger groups. This trend was consistent across all three frequencies of head actuation, and there was no difference between males and females. The damping ratio showed some variability with slightly higher values for the older groups, but no clear trend. These results are consistent with prior MRE studies quantifying the effect of age and sex on material properties at 50 Hz actuation [64]. The brain has been reported to decrease in shear stiffness at a rate of up to 1.0% per year, with a regionally heterogeneous response and some regions exhibiting up to 20% lower stiffness in older adults compared to younger adults [62, 66, 67].

### Group-Average Brain Deformation

The group-average strain response from tMRI was only compared across age due to the low sample sizes and a previous analysis showing minimal effects with respect to sex on the response [47]. The tMRI experiments have a stronger dependence on the input experimental kinematics of the rotational device and thus showed larger subject-to-subject variance in the strain response that is confounded by kinematics. However, the average peak angular velocity and acceleration were similar between groups (Fig. 7). The maximum strain showed a general decrease in the older groups relative to the younger ones, with a similar magnitude of decrease in 50th and 95th percentile strains across the three groups and a similar spatial distribution of strain. This result is contrary to what was initially expected from the material property results, given that a decrease in stiffness could theoretically result in an increase in strain. The maximum displacement results were more nuanced, with similar displacement fields for the young and mid-age groups, and lower displacement in the cortex of the cerebrum for the older adult group. This difference leads to a smaller gradient of displacement across the cortex and inwards into the white matter, leading to smaller strain magnitudes and less strain at the peripheries. Although the sample size of the older adult group is low (*n* = 5), the strain result was consistent with low MPS standard deviations (see supplementary material).

The differences in displacement are likely due to differences in the brain–skull interface, both in the size of the subarachnoid space (see Fig. 3) as well as its material properties. While the current dataset does not quantify the mechanics of this interface, it has been shown to be important in determining the brain’s biomechanical response. Using MRE, a study found changes in brain–skull interface properties with age that caused higher cortical strain in older adults [68]. The dura has been reported to be more tightly adhered to the skull with older age and bridging veins have been found to stiffen with age, indicating expected tighter coupling and higher strains [69]. In this study, the opposite trend was found with decreased displacement and strains at the periphery. This observation is limited by the small sample size of the older adult group (*n* = 5) for the tMRI experiments and by variability among subjects in head positioning and peak rotational kinematics. However, the apparent trend warrants further study using impulsive tMRI loading across ages and especially in older adults.

### Stratification of Groups

The choice of age cutoff for the group-average responses in this study were informed by prior literature quantifying structural and material property changes in the healthy brain. General trends indicate that the brain volume generally increases until adulthood (18 years old), stays relatively consistent until 40–50 years old, and then experiences atrophy [70]. During this time, the whole body growth of individuals (height and weight) occurs until the age of 20 years old [71]. Similarly MRE shear stiffness has been shown to decline throughout the lifespan [66], but experience an onset of rapid regional decrease after age 50 [67]. The range and standard deviations of the structural MRI, MRE, and tMRI group-average results show that the cohorts chosen in this study may be better clustered based on different cutoff ages or other factors. These age cutoffs were chosen *a priori* in the current study, but there remains future work to better stratify cohorts based on external features, such as age, stature, or head anthropometry. These cutoffs could also be chosen based on internal metrics, such as brain morphometry or material properties. Such studies will help inform which factors contribute most to the brain deformation response and create group-average responses and models based on these factors.

### Trends in FE Models Versus the Experiments

There have been recent advances in subject-specific modeling to include neuroimaging data, such as MRI and MRE, with the goal of creating frameworks for accurate digital twins and a precision medicine approach [12, 29, 30, 72]. The needs of research and industry in safety testing, however, still require the use of representative models for cohorts of individuals in specific impact or injury scenarios. Evaluations of these models are typically done against experimental brain deformation data for a subject-specific response or to multiple datasets. If the deformation response does not match, inverse calibration or parameterization could be done to improve the response. The range of 95th percentile MPS for the models was 0.0541–0.0637, with similar small differences of 20–25% for the 50th percentile MPS and volume fractions among the groups. The models also did not show a dependence on age, though males had slightly larger strains, likely due to the larger brain volumes. While the model strains were generally larger than the group-average response, calibration with stiffer linear viscoelastic material properties or a hyperviscoelastic material would likely lead to an improved response. In this study, however, instead of a comparison to the specific strain field, the trends of differences in strain response among the groups for the models and the group-average tMRI response were assessed. The group-average tMRI responses showed a consistent decrease with age among the groups for all strain metrics (Table 4). This decrease was also evident in the average data from all individual subjects. The mismatch in strain difference with age for the model likely points to other factors that need to be altered in the model, such as the modeling assumptions of fluid-filled regions like the ventricles and subarachnoid space. These same factors, most notably the brain–skull interface, may explain why the older adult groups have lower strains and why the models do not show the same trend. In addition to comparisons to the magnitude and distribution of strain, there is a need for future model development and refinement to consider validating the model response to the trends in brain deformation across individuals and groups.

### Utility of Brain Biomechanics Data in the Small Strain Regime

While the experimental MRI data presented in this study are in the small strain regime (0.1–5% strain), it serves as an important calibration and evaluation target for computational brain models. While some data are available at higher severity loading conditions [19, 20], it is obtained from post-mortem subjects and only includes brain motion and not strain. There are obvious tradeoffs with post-mortem tissue that in vivo data, albeit at smaller strain, can provide similarly valuable and complementary information for TBI prediction. Additionally, mechanical tests of the brain (measured ex vivo or in vitro) have exhibited nonlinearity in the stress–strain response [73], but the response is only mildly nonlinear, especially under shear deformation, in the regime spanning tMRI (5% strain) and concussion (approximately 20% strain [7]).

Since the brain must deform in the small strain regime on its way to large and injurious deformation, any simulation that aims to capture large deformation should also capture small deformations. We have previously shown the use of this low-strain in vivo data to evaluate strain across loading regimes, complementary to ex vivo data. To validate strain similarity and consistency across an order of loading magnitude, the distribution of MRE (~0.1% strain) and tMRI (~5% strain) deformation have been shown to be qualitatively and quantitatively similar [32]. Computational modeling efforts have also shown that a complementary combination of in vivo MRE data and ex vivo data can be used to tune various linear and nonlinear constitutive parameters, which are then successfully evaluated against low and high loading brain deformation experiments [12, 29, 30].

## Summary and Conclusion

Group-average responses were created from a large brain biomechanics neuroimaging dataset, including neuroanatomy from structural MRI, material properties from MRE, and brain deformation response from tMRI. The results showed a dependence of the brain’s structural and biomechanical changes on age, with some sex differences in brain volumes. In particular, the older adult group had lower brain volume, lower shear stiffness, and lower maximum strains. FE models created from the average responses of the age and sex groups showed some subtle differences between groups, but these were not consistent with the larger trends with age seen in the experimental data. There is a need for the collection of more data, especially brain deformation data, to mechanistically understand the changes in strain response with age, and for computational models to better match the trends observed across the group-average responses.

## Supplementary Information

Below is the link to the electronic supplementary material.

Supplementary file1 (PDF 551 kb)
