# Supplementary material for: Average Biomechanical Responses of the Human Brain Grouped by Age and Sex
Source: Ann Biomed Eng. 2025 Apr 9;53(6):1496–511. doi: 10.1007/s10439-025-03725-y (PMC12075284; doi:10.1007/s10439-025-03725-y)
Supplement: Supplementary file 1 — Supplementary file1 (PDF 551 kb) [file 10439_2025_3725_MOESM1_ESM.pdf]

Group Average Biomechanical Response of the Brain

SUPPLEMENTARY MATERIAL

Table S1: Calibrated linear viscoelastic material properties for the six groups using the group-average MRE data from Section 3.2. A detailed explanation of the calibration scheme and associated Prony series parameters can be found in in Alshareef et al. (2021) [12].

		G0 (Pa)	G1 (Pa)	t1 (ms)	G2 (Pa)	t2(ms)	G3 (Pa)	t3(ms)	Ginf (Pa)
**Young Male**	Cerebellum Gray	5535	322	48.6	508	6.4	3877	0.3	828
	Cortical Gray	4427	274	61.6	471	13.9	2763	1.1	920
	Deep Gray	10994	534	48.5	842	6.9	8268	0.3	1350
	Brainstem	2435	388	22.7	692	1.3	705	1.3	650
	Cerebellum White	7189	381	46.4	601	7.2	5179	0.3	1027
	Cerebrum White	5235	749	21.6	3388	1.0	n/a	n/a	1098
**Young Female**	Cerebellum Gray	6060	299	53.3	506	5.8	4555	0.3	700
	Cortical Gray	5480	367	60.7	554	7.5	3585	0.6	973
	Deep Gray	8917	685	35.0	797	6.1	6131	0.4	1303
	Brainstem	4120	352	39.5	518	7.8	2263	0.6	988
	Cerebellum White	4614	633	16.1	2984	0.7	n/a	n/a	997
	Cerebrum White	5727	737	21.5	3745	1.0	n/a	n/a	1245
**Mid-Age Male**	Cerebellum Gray	7058	364	56.8	639	5.0	5285	0.2	769
	Cortical Gray	4569	683	20.8	2877	1.1	n/a	n/a	1009
	Deep Gray	6042	915	22.2	3691	1.1	n/a	n/a	1436
	Brainstem	3639	290	42.4	401	8.5	2082	0.7	868
	Cerebellum White	4595	329	71.2	467	12.0	2788	1.0	1010
	Cerebrum White	5625	803	21.6	3642	1.0	n/a	n/a	1179
**Mid-Age Female**	Cerebellum Gray	5081	344	47.0	494	5.6	3561	0.4	682
	Cortical Gray	4571	282	79.1	482	12.4	2865	0.9	943
	Deep Gray	8965	626	36.7	772	7.0	6358	0.4	1208
	Brainstem	5339	323	45.3	461	6.0	3591	0.3	964
	Cerebellum White	7867	470	42.1	615	5.6	5883	0.3	898
	Cerebrum White	6898	430	60.5	634	7.6	4716	0.6	1118

**Older Male**	Cerebellum Gray	3762	291	53.6	403	7.7	2208	0.7	860
	Cortical Gray	4022	255	66.3	418	13.1	2581	1.0	769
	Deep Gray	5411	815	22.2	3324	1.1	n/a	n/a	1272
	Brainstem	2674	440	23.0	1460	1.3	n/a	n/a	775
	Cerebellum White	4179	676	22.1	2355	1.3	n/a	n/a	1148
	Cerebrum White	4852	683	21.6	3181	1.0	n/a	n/a	988
**Older Female**	Cerebellum Gray	5399	340	46.3	545	5.8	3829	0.3	685
	Cortical Gray	3963	575	21.7	2531	1.1	n/a	n/a	858
	Deep Gray	8938	501	43.3	731	6.1	6471	0.3	1234
	Brainstem	2539	406	21.8	1460	1.2	n/a	n/a	673
	Cerebellum White	4462	627	23.2	2766	1.1	n/a	n/a	1069
	Cerebrum White	4993	286	90.2	502	13.7	3245	0.9	959

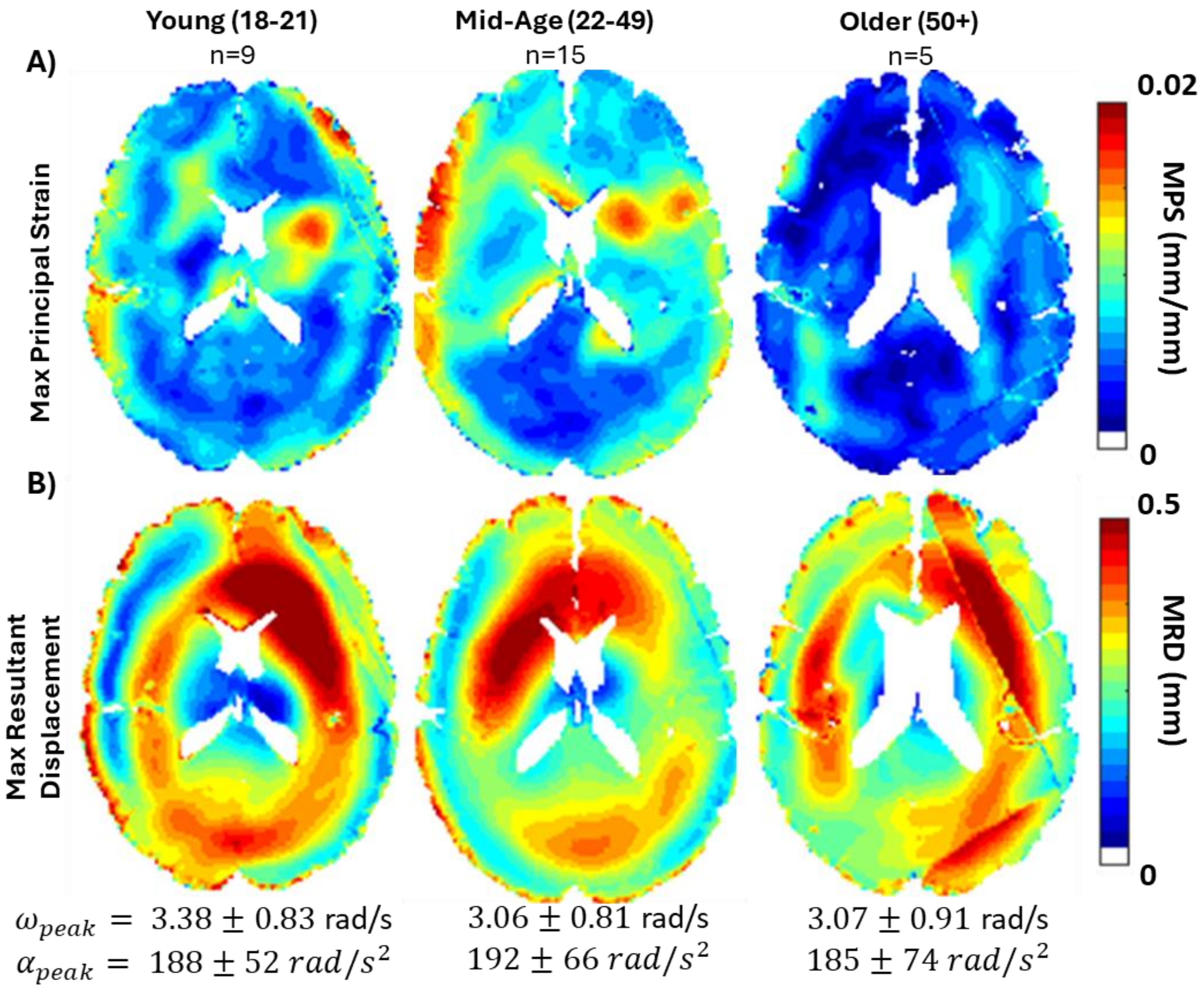

Figure S1: The standard deviation of (A) maximum principal strain (MPS) and (B) maximum resultant displacement from tagged MRI experiments were computed for the three age groups and are shown for an axial slice of the brain. Note that discontinuities in the displacement and strain fields for the older group-average result from inconsistent MRI field of view during acquisition.
